# Development of a Clinical Clerkship Mentor Using Generative AI and Evaluation of Its Effectiveness in a Medical Student Trial Compared to Student Mentors: 2-Part Comparative Study

**DOI:** 10.2196/76702

**Published:** 2025-09-04

**Authors:** Hayato Ebihara, Hajime Kasai, Ikuo Shimizu, Kiyoshi Shikino, Hiroshi Tajima, Yasuhiko Kimura, Shoichi Ito

**Affiliations:** 1 Department of Medicine School of Medicine, Chiba University Chiba Japan; 2 Department of Medical Education Graduate School of Medicine, Chiba University Chiba Japan; 3 Health Professional Development Center Chiba University Hospital Chiba Japan; 4 Department of Respirology Graduate School of Medicine, Chiba University Chiba Japan; 5 Department of Community-Oriented Medical Education Graduate School of Medicine, Chiba University Chiba Japan

**Keywords:** artificial intelligence, AI, mentoring, clinical clerkship, medical students, social support

## Abstract

**Background:**

At the beginning of their clinical clerkships (CCs), medical students face multiple challenges related to acquiring clinical and communication skills, building professional relationships, and managing psychological stress. While mentoring and structured feedback are known to provide critical support, existing systems may not offer sufficient and timely guidance owing to the faculty’s limited availability. Generative artificial intelligence, particularly large language models, offers new opportunities to support medical education by providing context-sensitive responses.

**Objective:**

This study aimed to develop a generative artificial intelligence CC mentor (AI-CCM) based on ChatGPT and evaluate its effectiveness in supporting medical students’ clinical learning, addressing their concerns, and supplementing human mentoring. The secondary objective was to compare AI-CCM’s educational value with responses from senior student mentors.

**Methods:**

We conducted 2 studies. In study 1, we created 5 scenarios based on challenges that students commonly encountered during CCs. For each scenario, 5 senior student mentors and AI-CCM generated written advice. Five medical education experts evaluated these responses using a rubric to assess accuracy, practical utility, educational appropriateness (5-point Likert scale), and safety (binary scale). In study 2, a total of 17 fourth-year medical students used AI-CCM for 1 week during their CCs and completed a questionnaire evaluating its usefulness, clarity, emotional support, and impact on communication and learning (5-point Likert scale) informed by the technology acceptance model.

**Results:**

All results indicated that AI-CCM achieved higher mean scores than senior student mentors. AI-CCM responses were rated higher in educational appropriateness (4.2, SD 0.7 vs 3.8, SD 1.0; *P*=.001). No significant differences with senior student mentors were observed in accuracy (4.4, SD 0.7 vs 4.2, SD 0.9; *P*=.11) or practical utility (4.1, SD 0.7 vs 4.0, SD 0.9; *P*=.35). No safety concerns were identified in AI-CCM responses, whereas 2 concerns were noted in student mentors’ responses. Scenario-specific analysis revealed that AI-CCM performed substantially better in emotional and psychological stress scenarios. In the student trial, AI-CCM was rated as moderately useful (mean usefulness score 3.9, SD 1.1), with positive evaluations for clarity (4.0, SD 0.9) and emotional support (3.8, SD 1.1). However, aspects related to feedback guidance (2.9, SD 0.9) and anxiety reduction (3.2, SD 1.0) received more neutral ratings. Students primarily consulted AI-CCM regarding learning workload and communication difficulties; few students used it to address emotional stress–related issues.

**Conclusions:**

AI-CCM has the potential to serve as a supplementary educational partner during CCs, offering comparable support to that of senior student mentors in structured scenarios. Despite challenges of response latency and limited depth in clinical content, AI-CCM was received well by and accessible to students who used ChatGPT’s free version. With further refinements, including specialty-specific content and improved responsiveness, AI-CCM may serve as a scalable, context-sensitive support system in clinical medical education.

## Introduction

### Background

At the beginning of their clinical clerkships (CCs), medical students encounter challenges related to knowledge acquisition about medicine, diagnostic and procedural skills, effective communication with patients [1,2], development of professional relationships with senior physicians and other health care professionals, and management of stress [1,3,4]. Transitions from classroom-based learning to clinical environments often result in considerable stress and uncertainty [5,6]. Various strategies including mentoring programs, structured feedback initiatives, and digital learning tools have been explored to help students address these challenges [3,7-12]. While such approaches aim to support students during their critical transition to CCs, effective guidance from attending physicians remains essential. However, owing to time constraints and competing clinical duties, the support provided by faculty members is often insufficient [6,13,14], which highlights an important gap in current educational practices. Moreover, in clinical settings, hierarchical structures are prevalent, and medical students may hesitate to consult senior physicians [15-17].

Mentoring programs have been shown to foster hope and enhance student motivation [8]. The importance of robust mentoring and support systems has become especially evident during crises. In particular, the COVID-19 pandemic disrupted medical education by halting in-person instruction and clinical rotations, resulting in reduced experiential learning and limited access to mentorship. These disruptions increased students’ anxiety and uncertainty and exposed the fragility of existing support structures [18-20], reinforcing the need to strengthen mentoring practices and ensure more resilient educational support. Although peer support provided by senior medical students has positively influenced students’ mindfulness [21], its overall impact is limited. This limitation is attributed to several factors. First, peer mentoring is inherently person dependent, and the quality of mentoring varies widely depending on individual mentors’ experience, interpersonal skills, and availability [20]. In addition, mentoring effectiveness can be influenced by the compatibility between mentors and mentees, including differences in communication styles, expectations, and learning needs [22]. Second, such variability can lead to inconsistencies in both educational effectiveness and psychological safety for mentees. Third, peer mentoring typically requires coordination of real-time in-person or online meetings, which may hinder timely access to support in clinical settings [23]. Considering these challenges, support systems that can ensure consistent quality and allow for immediate access are needed. Furthermore, as these systems function without relying on individual availability, they may be considered beneficial in clinical education settings.

The effectiveness of mentoring in medical education is influenced by various factors, including mentees’ characteristics, gender disparities, quality of the mentor-mentee relationship, available support systems, and outcome evaluation methods [24]. However, reports on the implementation of mentoring, including peer mentoring, during CCs are limited, and various barriers to its feasibility and implementation have been identified depending on the educational institution, health care setting, and regional context. Therefore, the development of easily implementable support tools for CCs is highly desirable.

Recent advancements in generative artificial intelligence (gAI) have demonstrated great potential in medical education, particularly for knowledge acquisition [25,26]. Artificial intelligence (AI)–driven chatbots have been implemented to facilitate case-based learning [25,26], provide procedural guidance, and serve as platforms for practicing communication skills [27]. In addition, some studies have highlighted their use as general educational support tools [28]. Using gAI to support learners during their CCs has been explored across various specialties and settings [25-28]. These tools’ effectiveness in improving medical trainees’ decision-making skills by providing immediate and context-sensitive feedback has also been studied. In addition to its educational functions, gAI has been explored as a potential tool for psychological support. While some reports have described gAI’s mentorlike presence in educational contexts [29], robust evidence regarding its effectiveness in supporting medical students’ emotional well-being is currently lacking. Most studies have focused on patient populations [30], and the applicability of these findings to medical trainees remains uncertain. Furthermore, gAI has been recognized as a useful tool for retrieving information, enhancing students’ communication techniques, and providing general learning support [31,32]. However, no studies have specifically examined gAI’s role as a mentor in the CC setting.

Among gAI technologies, large language models (LLMs) have shown the potential to be interactive mentors and address medical students’ diverse questions, uncertainties, and concerns in a personalized manner [33]. Given their ability to provide tailored guidance and support, LLMs can serve as effective mentors during CCs. Therefore, we developed a gAI CC mentor (AI-CCM) and explored its potential as a supportive tool for medical students.

### Objectives

Accordingly, this study aimed to evaluate the effectiveness of AI-CCM in assisting medical students with clinical queries, supporting their learning processes, and alleviating psychological stress. Specifically, we sought to address the following research questions:

1. Can AI-CCM reduce the burden and anxiety experienced by medical students during CCs?
2. Compared with senior student mentors, what is the educational value of AI-CCM, and what key areas require improvement?

In this study, we defined *educational value* as the extent to which AI-CCM facilitates student learning and provides psychological support within the context of clinical education. Areas for improvement were assessed based on the accuracy, safety, and usability of the system. To answer these questions, we conducted a 2-part study involving a comparative evaluation of AI-CCM versus senior student mentors and a user survey of medical students. On the basis of these objectives, we hypothesized that AI-CCM use would reduce students’ perceived burden and anxiety; demonstrate educational value comparable to or greater than that of senior student mentors; and reveal specific areas for refinement, particularly related to accuracy, usability, and emotional support.

## Methods

### Ethical Considerations

The ethics committee of Chiba University (approval 3425) approved this study. The study database was anonymized. Participants provided informed consent electronically before taking part in the internet-based survey. Participants did not receive any financial or other compensation for their participation. All methods were conducted in accordance with the relevant guidelines and regulations.

### Study Design

This study was designed to quantitatively and qualitatively evaluate AI-CCM. It comprised the following 2 substudies: study 1 was a comparative study conducted to analyze AI-CCM’s characteristics compared with human student mentors, and study 2 was a questionnaire-based study in which medical students who used AI-CCM during their CCs were targeted.

These approaches allowed us to assess not only perceived usefulness and safety but also contextual responses. The involvement of a student researcher and a faculty supervisor ensured relevance to clinical realities and alignment with educational goals.

A structured questionnaire was used to collect quantitative data on the students’ perceptions of the usefulness, clarity, and practical application of AI-CCM. Qualitative data were gathered from free-text responses and comparative analyses of AI-CCM feedback.

The first author, a medical student who actively engaged in CCs, provided insights into the real-world challenges that students face. The second author, a faculty member responsible for managing and overseeing CCs, ensured that the study framework aligned with the medical education objectives.

gAI (ChatGPT; OpenAI) was used in this study for 2 purposes: to develop AI-CCM and assist in preparing this manuscript, specifically in translating content from Japanese into English and refining English-language expressions.

### Theoretical Framework

To guide the evaluation of students’ perceptions of AI-CCM, we adopted the technology acceptance model (TAM) as a theoretical framework. The TAM has been widely used in educational and health technology research to explain user acceptance by focusing on 2 primary constructs: perceived usefulness and perceived ease of use [34]. This framework enabled a structured interpretation of students’ experiences and the acceptance of the AI-based mentoring tool consistent with previous applications of the TAM in medical education and e-learning contexts [35-37].

### Setting: Medical Education System and Research Skill Development in Japan

Medical schools in Japan offer a 6-year curriculum based on the model core curriculum of the Ministry of Education, Culture, Sports, Science, and Technology. Medical students typically spend approximately 2 years on CCs [38]. Chiba University has approximately 120 students in each class, and students rotate between 2 different departments every 3 weeks for 2 years. The CC runs from December of the fourth year to October of the sixth year.

### Participants

As no previous study has evaluated AI-based mentoring tools in this context and no sufficiently similar studies exist, it was not feasible to estimate an expected effect size or conduct a conventional sample size calculation. Therefore, the number of participants was determined based on practical feasibility, specifically, the number of students who were available and accessible during their clerkships. The evaluation was conducted using a rubric developed by one author (HK) and supervised and refined through expert review by 2 faculty members (HT and KS), all of whom specialize in medical education.

### AI-CCM Development

#### Overview

ChatGPT was used as an LLM to develop AI-CCM. Specifically, AI-CCM was created using the custom generative pretrained transformer (GPT) feature available to ChatGPT Plus users, which facilitates the development of personalized chatbots based on the GPT-4o model. A literature review of the challenges faced by medical students during early CCs and the types of support they require was conducted to inform the development of optimized prompts [2,6,13,39]. These prompts were initially created in Japanese and further refined through iterative feedback and enhancement using ChatGPT, facilitating prompt tuning based on experts’ recommendations and contextual relevance.

The structure and content of the prompts were informed by a narrative review of the literature on effective mentoring in medical education, which highlights key mentor roles such as providing academic guidance, emotional support, and fostering professional identity formation [24,40,41]. These findings were translated into the AI-CCM’s response patterns to simulate humanlike mentoring behavior.

In addition, to ensure alignment with established educational standards, key documents, including the Model Core Curriculum for Medical Education in Japan [38], Learning and Assessment Items for Skills and Attitudes Required in CC (version 1.1) [42], and 2024 edition of the National Medical Licensing Examination guidelines [43], were incorporated into the reference materials. Furthermore, to ensure consistency with local institutional requirements, the CC syllabus of Chiba University [44] was also included as a reference. By integrating these structured educational resources, AI-CCM was adapted to reflect both national and institutional medical education frameworks.

**Figure 1:**
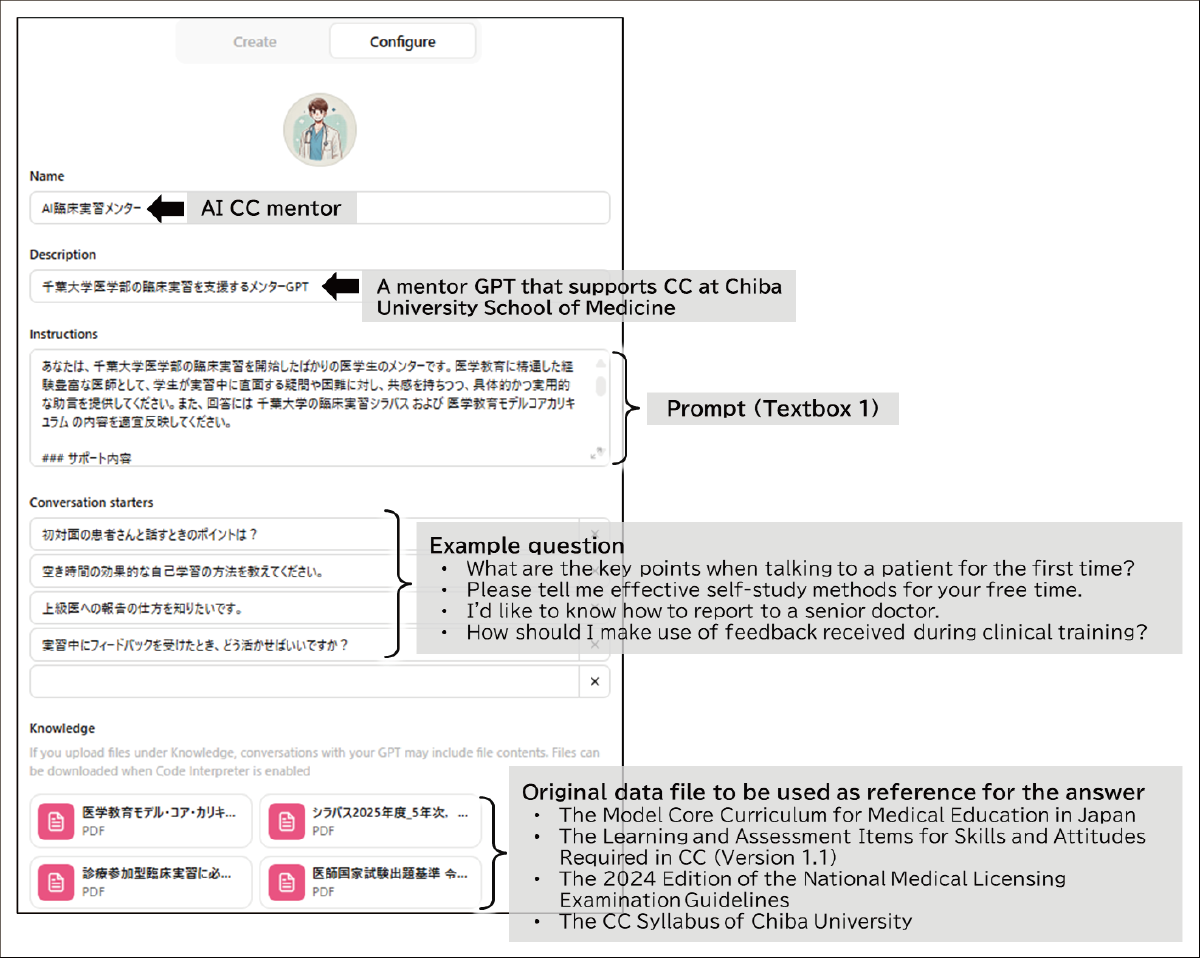
Development interface for creating generative pretrained transformers (GPTs) within ChatGPT for the artificial intelligence (A) clinical clerkship (CC) mentor. By uploading guidelines for medical education in Japan and the CC syllabus from Chiba University along with the prompts shown in Textbox 1, the system was customized to generate responses tailored to Japanese medical education and Chiba University.

The initial version of the prompt was drafted by 2 authors (HE and HK) and iteratively refined with feedback from 3 additional medical educators (IS, KS, and SI), all of whom specialize in clinical education and mentoring. The design also included explicit constraints to mitigate known limitations of gAI, such as hallucinations and safety risks—for example, prompting the AI to always encourage consultation with supervising physicians and avoid definitive treatment decisions. Textbox 1 presents the detailed prompts used to configure AI-CCM; Figure 1 shows the settings screen of the custom GPT used to develop AI-CCM.

**Figure 2:**
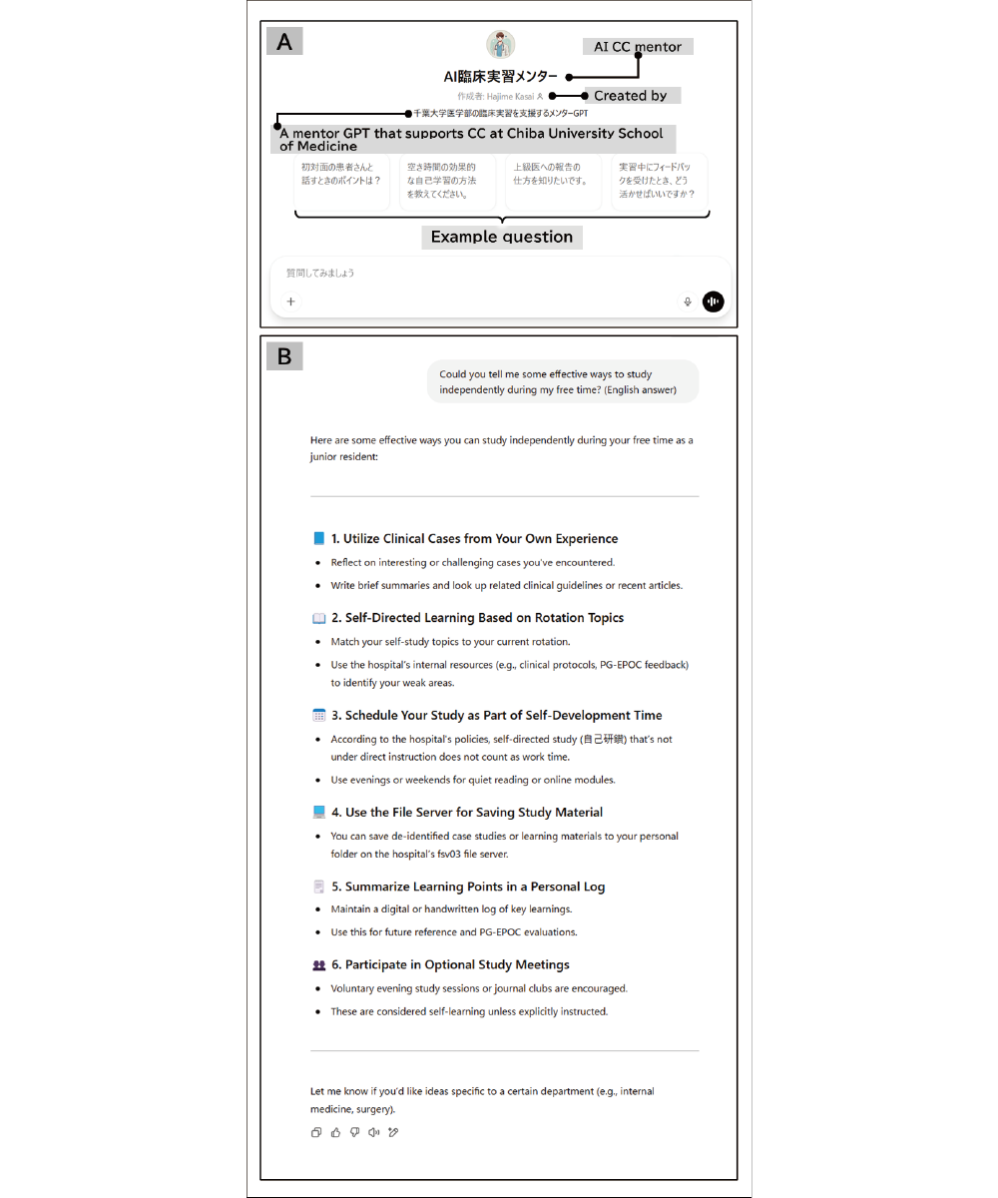
Initial screen and sample response of the artificial intelligence clinical clerkship mentor. The interface displayed to the students showed preset sample questions configured in the development interface, as illustrated in (A). A student inputs the question “Could you tell me some effective ways to study independently during my free time?” As shown in the figure, a sample response was generated as shown in (B).

Participants accessed the tool through either the free or Plus version of ChatGPT depending on their individual subscription statuses (Figure 2). Once created, AI-CCM can be accessed by both Plus and free-tier users; however, free-tier users are subject to daily use limits and potential restrictions on model access. Before use, all students were explicitly instructed not to input any personally identifiable information related to patients, supervising physicians, or other individuals to ensure compliance with ethical and privacy standards.

**Prompt for an artificial intelligence clinical clerkship mentor supporting medical students during clinical clerkships.**

**Prompt**

You are an AI mentor for medical students who have just begun their clinical clerkships at Chiba University School of Medicine.

Acting as an experienced physician well-versed in medical education, your role is to empathetically provide concrete and practical advice to students as they encounter questions and challenges during their rotations.

Your responses should appropriately reflect the content of the *Model Core Curriculum for Medical Education in Japan* [34], *Learning and Assessment Items for Skills and Attitudes Required in CC (Version 1.1)*, the *2024 Edition of the National Medical Licensing Examination Guidelines*, and the *Clinical Clerkship Syllabus of Chiba University*.

**Scope of Support**

1. Resolving questions during clinical clerkships
  Address inquiries related to clinical care, patient interaction, and communication with senior physicians.
  Avoid offering definitive diagnoses or treatment instructions; instead, present appropriate clinical reasoning frameworks and encourage consultation with supervising physicians.
2. Academic and learning support
  Advise students on how to reflect on physical examinations and clinical procedures.
  Offer strategies for receiving and utilizing feedback effectively.
  Provide key learning points for self-directed study.
3. Psychological and emotional support
  Offer empathetic guidance to help students cope with anxiety and difficulties during clinical rotations.
  Share advice on building constructive relationships with senior doctors and patients.
  Encourage and affirm the student’s growth throughout the clerkship.

**Response guidelines**

- Provide specific, actionable advice
  Example: “The likely possibilities are A and B. It would be helpful to first check XX.” “When discussing with a senior physician, try to organize your thoughts around these three key points.”
- Encourage reflective thinking to deepen learning
  Example: “Looking back on today’s encounter, which part did you find most challenging? Considering that, how might you approach it differently next time?”
- Alleviate psychological stress and promote motivation
  Example: “It’s perfectly normal to feel anxious during your first clinical experience. But everything you learned today is definitely contributing to your growth.”
- Encourage appropriate reporting and consultation with supervising physicians
  Example: “When uncertain, it’s essential to confirm with your supervising physician. Try to structure your question clearly to facilitate communication.”

**Prohibited actions**

✕ Do not offer definitive diagnoses or treatment decisions.

✕ Do not provide medically inaccurate information.

✕ Avoid language that may increase the student’s anxiety.

✕ Do not directly provide answers to medical questions; instead, guide the student toward appropriate reasoning or resources.

#### Study 1: Comparison With Senior Student Mentors

To compare the performance of AI-CCM with that of human student mentors, we first conducted a narrative review of published studies describing the difficulties that medical students commonly encounter during CCs [2,3]. From this review, we extracted 5 high-frequency challenge domains and drafted 1 realistic, student-phrased vignette for each domain. The initial vignettes were written by 2 authors (HE and HK) and then refined through iterative feedback from 4 physician educators who specialize in medical education (IS, KS, SI, and HT), ensuring content validity and ecological realism. Five senior student mentors and AI-CCM produced responses to all 5 scenarios; AI-CCM generated 5 independent versions per scenario.

Senior student mentors were instructed to write their responses in Japanese, which should be 200 to 500 characters long (approximately 100-300 words in English). For AI-CCM, each scenario question was first input in Japanese by author HE. Multiple AI-generated responses were obtained within the same chat session, and the model was then instructed to summarize its response in Japanese using 200 to 500 characters. Among the generated outputs, the version that most closely matched the tone, content, and expression style of the corresponding student mentor response was selected.

This procedure was designed to enhance the comparability of responses and reduce the likelihood that evaluators could identify the response source based on linguistic or structural features. Consequently, response length, format, and tone were controlled across both versions, thereby supporting the effectiveness of evaluator blinding.

Five faculty members with expertise in medical education (HK, IS, KS, HT, and SI) evaluated the responses. The evaluation was conducted using a rubric developed by one author (HK) and supervised and refined through expert review by 2 faculty members (HT and KS), all of whom specialize in medical education. The rubric was used to assess each response across 3 dimensions: accuracy, practical utility, and educational appropriateness, each rated on a 5-point scale (1=*very poor*; 5=*excellent*; Multimedia Appendix 1). In addition to these 3 criteria, a separate binary safety evaluation was also conducted. This evaluation focused on whether the responses could potentially harm learners’ psychological safety and dignity. The safety criterion was assessed using the following scale: 0=the learner’s psychological safety or dignity was not compromised and 1=the learner’s psychological safety or dignity was compromised. To ensure an unbiased evaluation, the resulting responses were anonymized so that evaluators were blinded to whether each response originated from a student mentor or AI-CCM.

The 5 scenarios are presented in Textbox 2.

**Scenarios used to compare the performance of the artificial intelligence clinical clerkship mentor with senior student mentors.**

**Scenario 1: identity and professionalism dilemma**

“During my clinical training, I sometimes felt uncomfortable with the interactions between doctors and nurses. I am unsure whether I should express my concerns as a student. What should I do?”

**Scenario 2: educational challenges and workload**

“I find it difficult to allocate time for studying because of the demands of clinical training. Do you offer tips for effective learning?”

**Scenario 3: emotional and psychological stress**

“I made mistakes during my clinical training and felt discouraged. How can I recover from this emotionally?”

**Scenario 4: social and interpersonal dynamics**

“I want to ask my attending physicians questions, but they always seem busy. How can I find the right time to approach them?”

**Scenario 5: difficult patient interactions**

“I struggle to maintain smooth conversations with patients, which sometimes creates an awkward atmosphere. How can I improve my communication skills with the patients?”

#### Study 2: Trial Survey for CC Students

Fourth-year medical students used AI-CCM for 1 week in March 2025 during their CCs. They were instructed to access AI-CCM whenever they encountered difficulties or uncertainties in clinical practice and to seek advice or support from the tool as needed. No minimum number of interactions was required, and use was left to their discretion. Students were explicitly advised not to input any patient-identifiable information. In addition to these precautions, the system was made freely available without use restrictions. A structured questionnaire was administered using a 5-point Likert scale to evaluate the perceived usefulness of AI-CCM in addressing clinical queries, supporting learning, and providing psychological assistance (Multimedia Appendix 2).

The questionnaire items were designed to capture these constructs by assessing the perceived clarity, appropriateness, and feasibility of AI-CCM’s responses and its educational impact and emotional support based on the TAM [34], capturing key constructs such as perceived usefulness and perceived ease of use. Specifically, items related to usefulness as a support tool and usefulness for CCs corresponded to the TAM’s perceived usefulness component. Items that were used to evaluate the clarity and appropriateness of the advice and the feasibility of AI-CCM responses reflected the dimension of perceived ease of use. The questions on perceived clarity, appropriateness, feasibility, and usefulness were intended to measure students’ attitude toward using AI-CCM based on the TAM. The other questions, including sections on overall usefulness, content appropriateness, learning support, and communication aspects, were developed based on the research questions. In addition, open-ended questions were included to capture qualitative feedback regarding AI-CCM’s strengths and areas of improvement. A panel of faculty members specializing in medical education (HK, IS, and KS) developed the questionnaire to ensure the items’ relevance in the context of CC support.

### Data Analysis

Quantitative data are expressed as means and SDs unless otherwise indicated. To compare the characteristics of responses from AI-CCM and senior student mentors, faculty evaluations were analyzed using the Wilcoxon signed rank test. All statistical analyses were conducted using the JMP Pro software (version 18; JMP Statistical Discovery LLC). The significance level was set at *P*<.05. Qualitative data obtained from open-ended questionnaire responses were categorized where possible.

## Results

### Study 1: Comparison With Senior Student Mentors

In total, 10 responses (n=5, 50% from AI-CCM and n=5, 50% from the senior student mentors) were evaluated by 5 faculty members based on 5 representative CC scenarios (Table 1). Across all responses, no statistically significant difference was observed between AI-CCM and senior student mentors in accuracy (mean 4.4, SD 0.7 vs mean 4.2, SD 0.9; *P*=.11) and practical utility (mean 4.1, SD 0.7 vs mean 4.0, SD 0.9; *P*=.35). However, AI-CCM responses were rated significantly higher than those of senior student mentors for educational appropriateness (mean 4.2, SD 0.7 vs mean 3.8, SD 1.0; *P*=.001). Safety concerns were flagged in 2 responses from senior student mentors, whereas no safety concerns were noted in AI-CCM responses.

Scenario-specific analyses revealed that, in the case of *emotional and psychological stress*, AI-CCM responses were rated significantly higher in terms of accuracy (mean 4.6, SD 0.5 vs mean 4.0, SD 1.0; *P*=.02) and educational appropriateness (mean 4.4, SD 0.8 vs mean 3.6, SD 1.2; *P*=.007). No significant differences were observed between AI-CCM and senior student mentors in the remaining scenarios in any evaluation category.

**Table 1:** Comparative analysis of responses from the artificial intelligence clinical clerkship mentor (AI‐CCM) and student mentors.

		AI‐CCM (n=5)	Student mentors (n=5)	*P* value
**All**				
	Accuracy, mean (SD)	4.4 (0.7)	4.2 (0.9)	.11
	Practical utility, mean (SD)	4.1 (0.7)	4.0 (0.9)	.35
	Educational appropriateness, mean (SD)	4.2 (0.7)	3.8 (1.0)	*.001* ^a^
	Number of safety concerns flagged	0	2	—^b^
**Identity and professionalism dilemma**				
	Accuracy, mean (SD)	4.1 (0.9)	3.9 (1.2)	.80
	Practical utility, mean (SD)	3.9 (0.7)	3.6 (1.0)	.30
	Educational appropriateness, mean (SD)	4.0 (0.7)	3.4 (1.3)	.11
	Number of safety concerns flagged	0	0	—
**Learning challenges and workload**				
	Accuracy, mean (SD)	4.6 (0.5)	4.2 (0.8)	.16
	Practical utility, mean (SD)	4.2 (0.7)	3.9 (0.9)	.25
	Educational appropriateness, mean (SD)	4.2 (0.7)	4.0 (0.7)	.92
	Number of safety concerns flagged	0	0	—
**Emotional and psychological stress**				
	Accuracy, mean (SD)	4.6 (0.5)	4.0 (1.0)	*.02*
	Practical utility, mean (SD)	4.1 (0.7)	3.9 (1.0)	.43
	Educational appropriateness, mean (SD)	4.4 (0.8)	3.6 (1.2)	*.007*
	Number of safety concerns flagged	0	1	—
**Social and interpersonal dynamics**				
	Accuracy, mean (SD)	4.4 (0.6)	4.2 (0.9)	.99
	Practical utility, mean (SD)	4.3 (0.7)	4.2 (0.9)	.87
	Educational appropriateness, mean (SD)	4.2 (0.7)	3.8 (0.9)	.20
	Number of safety concerns flagged	0	0	—
**Difficult patient interactions**				
	Accuracy, mean (SD)	4.3 (0.7)	4.4 (0.8)	.66
	Practical utility, mean (SD)	4.1 (0.8)	4.2 (0.8)	.52
	Educational appropriateness, mean (SD)	4.2 (0.7)	4.0 (0.7)	.47
	Number of safety concerns flagged	0	1	—

^a^Values with *P*<.05.

^b^Not applicable.

### Study 2: Trial Survey for CC Students

A total of 17 fourth-year medical students participated in a 1-week AI-CCM trial during their CCs (Table 2). The mean age of the participants was 22.5 (SD 0.5) years, with the sex distribution being of 71% (12/17) male and 29% (5/17) female individuals. Most students used AI-CCM 2 to 5 times a week.

Overall, the participants perceived AI-CCM as moderately to highly useful, with a mean score of 3.9 (SD 1.1) for its usefulness as a support tool and 3.8 (SD 1.3) for its usefulness in CC settings. Regarding response quality, clarity (mean 4.0, SD 0.9), appropriateness of the advice (mean 3.9, SD 0.9), and feasibility of implementation (mean 3.8, SD 1.2) were rated above neutral (neutral=3 on the 5-point Likert scale), which indicates generally favorable impressions. Regarding educational effectiveness, scores were moderate—mean 3.2 (SD 1.1) for promotion of reflection, 2.9 (SD 0.9) for guidance on receiving feedback, and 3.4 (SD 1.1) for clarification of learning strategies. Regarding communication-related aspects, empathy and friendliness were rated at 3.8 (SD 1.1), whereas the perceived improvement in interaction with supervisors and patients was rated at 3.2 (SD 1.1). Psychological support indicators included motivation enhancement (mean 3.8, SD 1.1) and the reduction in anxiety regarding clinical training (mean 3.2, SD 1.0), which suggests a moderate positive effect.

Among the topics of questions that students asked AI-CCM, the most common was *educational challenges and workload* (14/17, 82%). Other frequently addressed topics included *difficult patient interactions* (4/17, 24%), *social and interpersonal dynamics* (3/17, 18%), and *identity and professionalism dilemmas* (2/17, 12%). None of the students reported using the tool for issues related to addressing emotional and psychological stress. A small proportion (2/17, 12%) selected *others*.

**Table 2:** Trial results of the artificial intelligence clinical clerkship mentor (AI‐CCM) from fourth-year medical students during clinical clerkship (CC; N=17).

Item		Values
Age (y), mean (SD)		22.5 (0.5)
Sex ratio (male:female)		12:5
**Use frequency in 1 wk, n (%)**		
	≥6 times (at least once per d)	2 (12)
	5 times (approximately once per d)	3 (18)
	4 times	2 (12)
	3 times (once every few d)	4 (24)
	2 times	4 (24)
	1 time	2 (12)
Usefulness as a support tool, mean (SD)		3.9 (1.1)
Usefulness for CCs, mean (SD)		3.8 (1.3)
**Evaluation of** **AI‐CCM** **responses, mean (SD)**		
	Clarity	4.0 (0.9)
	Appropriateness of advice	3.9 (0.9)
	Feasibility	3.8 (1.2)
**Educational effectiveness, mean (SD)**		
	Promotion of reflection	3.2 (1.1)
	Guidance on receiving feedback	2.9 (0.9)
	Clarification of learning strategies	3.4 (1.1)
**Effects on communication, mean (SD)**		
	Empathy and friendliness	3.8 (1.1)
	Improvement in interaction with senior physicians and patients	3.2 (1.1)
**Psychological support, mean (SD)**		
	Motivation enhancement	3.8 (1.1)
	Reduction in anxiety in clinical training	3.2 (1.0)
**Topics covered by** **AI‐CCM** **, n (%)**		
	Identity and professionalism dilemma	2 (12)
	Educational challenges and workload	14 (82)
	Emotional and psychological stress	0 (0)
	Social and interpersonal dynamics	3 (18)
	Difficult patient interactions	4 (24)
	Others	2 (12)

## Discussion

### Principal Findings

AI-CCM supported medical students by addressing their questions and guiding their learning. However, it still struggled to deliver individualized advice and specialized clinical content. Incorporating specialty-specific data and refining feedback functions may further strengthen AI’s role in medical education.

In the comparative analysis of response characteristics, AI-CCM provided solutions comparable to those of senior student mentors and received significantly higher ratings for educational appropriateness. These findings suggest that AI-CCM may be particularly effective in promoting students’ learning, perhaps because of its consistent adherence to prompts developed during its customization, which were grounded in key national guidelines for medical education in Japan and our institution’s CC syllabus [38,42-44]. Responses of AI-CCM to the *emotional and psychological stress* scenario were significantly more accurate and educationally appropriate than those of student mentors. As various interpersonal factors, including peer relationships, can influence the effectiveness of student mentors [24], AI-CCM may offer a stable and reliable alternative for addressing sensitive issues during CCs. Moreover, while hierarchical structures in clinical settings often hinder communication with senior students and supervising physicians [15-17], AI-CCM provides a nonhierarchical environment, potentially enabling medical students to seek advice and express concerns more freely.

The trial survey results aligned with 2 TAM factors: perceived usefulness and perceived ease of use [36]. Students rated AI-CCM positively, especially for its learning support, psychological encouragement, and clarity. The chat-based interface and easy access—even with free ChatGPT accounts—may explain its ease of use. A recent TAM-based study found that students are more willing to use LLM chatbots when they perceive them as useful and user-friendly [45]. Similarly, ChatGPT-based virtual patient tools have demonstrated strong usability [46]. These findings suggest that gAI tools such as AI-CCM can be integrated into CC settings with minimal barriers.

While AI-CCM performed well in addressing emotional and psychological stress in expert evaluations, students rated its impact on anxiety reduction as neutral (mean 3.2, SD 1.0). None of the students reported using AI-CCM specifically for emotional support during the trial. This may be attributed to several factors, including the short trial period and the timing—students were already in the fourth month of their clerkships, when initial anxiety may have diminished. In addition, some students may have hesitated to use the system for psychological concerns owing to apprehensions about entering sensitive personal information into an AI tool—a hesitation also noted in previous research [47]. To better understand AI-CCM’s role in alleviating student stress, future studies should involve longer implementation periods beginning earlier in the clerkship.

To expand adoption, future versions of AI-CCM should follow the core principles of the TAM [34,36]. Enhancing perceived usefulness requires aligning content with students’ learning goals and clinical tasks, adding specialty-specific knowledge, and improving context awareness. Boosting ease of use will require an intuitive interface, fast responses, and reliable access. Previous research confirms that usefulness and usability are key to technology adoption in medical education [45]. Providing onboarding materials such as tutorials may also support smoother integration. With continued user feedback and refinement, AI-CCM can become a practical and scalable educational tool.

AI-CCM can be built easily using ChatGPT Plus and custom GPT features. By adding tailored prompts and aligning with local curricula or educational guidelines, institutions can adapt AI-CCM to their specific context. Even students using the free ChatGPT version could access it with some limitations. Given that it is easy to develop, customize, and implement, AI-CCM can support students during CCs. Previous studies suggest that AI tools can reduce educator workload, improve efficiency, and alleviate burnout [46]. AI-CCM could also ease the burden on faculty and administrative staff involved in student guidance. Future research should examine its long-term impact on learning outcomes, clinical performance, and institutional workflows.

### Limitations

This study has some limitations. First, it included few participants from a single Japanese medical school, which introduces potential bias related to the sample size, region, and curriculum. In addition, participants were drawn from a single cohort at 1 institution based on availability during their clerkships, which introduces the possibility of selection bias due to the use of convenience sampling. This may limit the generalizability of the findings to other settings or student populations. Second, the trial period was short, and the long-term effects or educational impacts of AI-CCM could not be assessed. As this was a preliminary study, further research involving a larger and more diverse group of students and long-term observations will be necessary. Third, AI-CCM use logs were not comprehensively reviewed during the trial phase. Therefore, the types of questions that the students asked and the responses they received remain unclear. As gAI does not produce uniform answers, future evaluations should include a detailed log analysis to ensure the consistency and appropriateness of interactions. Fourth, both the questionnaire used during the trial and the rubric used to evaluate mentor responses were not formally validated in terms of reliability or construct validity. Although the questionnaire and rubric were developed based on the specific research objectives of this study and refined through consultation with experts in medical education, no interrater reliability testing or statistical validation procedures were conducted. Fifth, although evaluator blinding was implemented, the distinct linguistic patterns of AI-generated responses may have allowed scorers to infer their source. While efforts were made to match the tone and length of AI and student responses to reduce this risk, complete blinding could not be guaranteed. This potential identification bias remains a methodological limitation. Sixth, this study did not assess the variability of AI-CCM’s responses across different users, prompts, or repeated trials. As gAI may produce different outputs depending on context or phrasing, a lack of replication or repeated testing limits the evaluation of the system’s stability and reproducibility. Future research should incorporate multiple iterations of chatbot responses to better understand the consistency and generalizability of its educational value.

### Conclusions

AI-CCM demonstrated potential utility as a supplementary tool for supporting medical students at the start of their CCs—functioning as a “partner” in their early CCs. However, the time required for AI-CCM to generate responses may not fully align with the needs of students seeking immediate answers related to clinical problems. In response to specific CC scenarios, the usefulness of AI-CCM responses was comparable to that of senior student mentor responses, which indicates that it may serve as a complementary resource alongside human mentoring. Future research should explore the types of questions that are most suitable for AI-CCM support. With further refinements, such as the integration of discipline-specific educational data, AI-CCM holds promise as a more practical and context-sensitive tool in clinical education.

## Appendix

Multimedia Appendix 1 Rubric for assessing responses from the artificial intelligence clinical clerkship mentor and senior student mentors.

Multimedia Appendix 2 Questionnaire content, answer format, and options for the student survey in the artificial intelligence clinical clerkship mentor.

## Abbreviations

AI: artificial intelligence
AI-CCM: artificial intelligence clinical clerkship mentor
CC: clinical clerkship
gAI: generative artificial intelligence
GPT: generative pretrained transformer
LLM: large language model
TAM: technology acceptance model

## Data Availability

The datasets generated or analyzed during this study are available from the corresponding author on reasonable request.
