# Supplementary material for: Development of a Clinical Clerkship Mentor Using Generative AI and Evaluation of Its Effectiveness in a Medical Student Trial Compared to Student Mentors: 2-Part Comparative Study
Source: JMIR Med Educ. 2025 Sep 4;11:e76702. doi: 10.2196/76702 (PMC12447005; doi:10.2196/76702)
Supplement: Multimedia Appendix 1 [file mededu_v11i1e76702_app1.docx]

**Supplementary Table 1. Rubric for assessing responses from the AI-Clinical Clerkship Mentor and senior student mentors.**

| Evaluation criteria | 1 (Very bad) | 2 (Bad) | 3 (Moderate) | 4 (Good) | 5 (Very Good) |
| --- | --- | --- | --- | --- | --- |
| Accuracy | Contains incorrect or misleading information that may cause misunderstandings. | Includes some incorrect or ambiguous statements, making it less reliable. | Mostly accurate, but may contain slightly vague expressions or uncertain information. | Provides accurate information with minimal risk of misunderstanding. | All information is correct, clear, and completely free from misleading elements. |
| Practical Utility | Content is abstract or impractical, with little relevance to real clinical or training situations. | Some useful aspects, but lacks specificity, making it difficult to apply in real settings. | Generally applicable, but requires adaptation to specific situations. | Contrary to concrete advice that can be effectively used in clinical settings. | Provides clear and specific advice that can be immediately implemented in real clinical or training environments. |
| Educational Appropriateness | Lacks elements that encourage learning; explanation is one-sided or difficult to understand. | Supports learning but contains unclear aspects, making it hard for students to fully grasp. | Provides some learning support but lacks elements that promote deep thinking. | Encourages student thinking and enhances learning effectiveness. | Actively promotes independent learning, incorporating appropriate questioning and feedback. |
