# Supplementary material for: Development of a Clinical Clerkship Mentor Using Generative AI and Evaluation of Its Effectiveness in a Medical Student Trial Compared to Student Mentors: 2-Part Comparative Study
Source: JMIR Med Educ. 2025 Sep 4;11:e76702. doi: 10.2196/76702 (PMC12447005; doi:10.2196/76702)
Supplement: Multimedia Appendix 2 [file mededu_v11i1e76702_app2.docx]

**Supplementary Table 2. Questionnaire content, answer format, and options for the student survey in the AI-CCM.**

| **Survey Item (English)** | **Response Format and Choices (English)** |
| --- | --- |
| Department of Clinical Rotation | Single choice: Gastroenterology / Endocrinology, Diabetes and Metabolism / Hematology / Cardiology / Cardiovascular Surgery / Pulmonology / Thoracic Surgery / Allergy and Rheumatology / Nephrology / General Medicine / Hepatobiliary and Pancreatic Surgery, Breast Surgery / Esophageal and Gastrointestinal Surgery, Breast Surgery, Transplant Surgery / Pediatrics / Obstetrics and Gynecology / Emergency Medicine and Intensive Care |
| Frequency of using AI‐CCM in one week | Single choice: 6 times or more (once or more per day) / 5 times (about once per day) / 4 times / 3 times (every few days) / 2 times / 1 time / Did not use |
| Overall Evaluation |  |
| The AI‐CCM mentor was useful as a support tool for CC. | 5-point Likert scale  (1=Strongly disagree to 5=Strongly agree) |
| The AI‐CCM mentor's responses were helpful in actual CC. | Same as above |
| Evaluation of AI‐CCM Responses |  |
| The AI‐CCM mentor's responses were practical and easy to understand. | 5-point Likert scale  (1=Strongly disagree to 5=Strongly agree) |
| The AI‐CCM mentor provided appropriate advice. | Same as above |
| The AI‐CCM mentor's responses included actionable suggestions. | Same as above |
| Educational Effectiveness |  |
| The AI‐CCM mentor helped me reflect on clinical skills. | 5-point Likert scale  (1=Strongly disagree to 5=Strongly agree) |
| The AI‐CCM mentor taught me how to receive effective feedback. | Same as above |
| Using the AI‐CCM mentor clarified how to proceed with my learning. | Same as above |
| Effects on Communication |  |
| The AI‐CCM mentor was empathetic and approachable. | 5-point Likert scale  (1=Strongly disagree to 5=Strongly agree) |
| Advice from the AI‐CCM improved my communication with senior doctors and patients. | Same as above |
| Psychological Support |  |
| Encouragement and advice from the AI‐CCM increased my motivation for the CC. | 5-point Likert scale  (1=Strongly disagree to 5=Strongly agree) |
| The AI‐CCM mentor reduced my anxiety toward CC. | Same as above |
| What kind of questions did you ask the AI‐CCM? | Multiple choice: Concerns about role as a student / Ethical dilemma in clinical settings / Balancing clinical practice and learning / Lack of clinical knowledge and skills / Coping with stress and pressure during clerkship / How to deal with mistakes and failures / Coping with patient deterioration or death / How to ask or consult supervisors / How to build relationships with supervisors / How to communicate and build relationships with patients / Others |
| Good points of the AI‐CCM | Free text |
| Negative points or aspects to improve | Free text |
| Functions or support to be added in the future | Free text |

AI‐CCM, AI Clinical Clerkship Mentor; CC, Clinical clerkship.
